# Nonlinearity-induced nanoparticle circumgyration at sub-diffraction scale

**DOI:** 10.1038/s41467-021-24100-0

**Published:** 2021-06-17

**Authors:** Yaqiang Qin, Lei-Ming Zhou, Lu Huang, Yunfeng Jin, Hao Shi, Shali Shi, Honglian Guo, Liantuan Xiao, Yuanjie Yang, Cheng-Wei Qiu, Yuqiang Jiang

**Affiliations:** 1 State Key Laboratory of Molecular Developmental Biology, Institute of Genetics and Developmental Biology, Chinese Academy of Sciences, Beijing, China; 2 Innovation Academy for Seed Design, Chinese Academy of Sciences, Beijing, China; 3 University of Chinese Academy of Sciences, Beijing, China; 4 Department of Electrical and Computer Engineering, National University of Singapore, Singapore, Singapore; 5 Department of Optical Science and Engineering, Hefei University of Technology, Hefei, Anhui China; 6 College of Science, Minzu University of China, Beijing, China; 7 State Key Laboratory of Quantum Optics and Quantum Optics Devices, Collaborative Innovation Center of Extreme Optics, Institute of Laser Spectroscopy, Shanxi University, Taiyuan, China; 8 School of Physics, University of Electronic Science and Technology of China, Chengdu, China

**Keywords:** Nanophotonics and plasmonics, Optical manipulation and tweezers

## Abstract

The ability of light beams to rotate nano-objects has important applications in optical micromachines and biotechnology. However, due to the diffraction limit, it is challenging to rotate nanoparticles at subwavelength scale. Here, we propose a method to obtain controlled fast orbital rotation (i.e., circumgyration) at deep subwavelength scale, based on the nonlinear optical effect rather than sub-diffraction focusing. We experimentally demonstrate rotation of metallic nanoparticles with orbital radius of 71 nm, to our knowledge, the smallest orbital radius obtained by optical trapping thus far. The circumgyration frequency of particles in water can be more than 1 kHz. In addition, we use a femtosecond pulsed Gaussian beam rather than vortex beams in the experiment. Our study provides paradigms for nanoparticle manipulation beyond the diffraction limit, which will not only push toward possible applications in optically driven nanomachines, but also spur more fascinating research in nano-rheology, micro-fluid mechanics and biological applications at the nanoscale.

## Abstract (web summary)

It has been challenging to rotate nanoparticles orbitally via optical trapping beyond the diffraction limit. Here, the authors take advantage of the nonlinear optical effect and demonstrate fast and controlled orbital rotation at subwavelength scale with a femtosecond pulsed Gaussian beam.

## Introduction

Optical manipulation has found applications in many research areas^1–3^, including biology^4–6^, atomic physics^7,8^, colloidal sciences^9^ and micro-fluidic physics^10,11^ by trapping and manipulating objects at the microscale and nanoscale. It’s still attracting great interests in various fundamental research^12–15^ and practical applications^16–19^. Besides manipulation and control of the objects with the trapping^1^, pushing^20^, pulling forces^21^ in the translation degree of freedom, the ability to rotate objects offers a new degree of control^22,23^. Rotation with controllable rotation speed, orbital radius and rotation direction has tremendous values in micromachines^24^ and metrology^25,26^. Among the strategies proposed to realize rotation of particles over the past decades^24–30^, utilizing the transfer from photonic angular momentum to particles is an appealing way. A trapped object can be rotated either by the transfer of spin angular momentum (SAM) from a circularly polarized beam, or by the transfer of orbital angular momentum (OAM) from a vortex beam. Assisted by SAM of the optical beam, the spin rotation frequency of the particle has reached several kHz in water^27,28^, several MHz in vacuum using the birefringent materials^25^ and even 5 GHz in vacuum using designed nano dumbbell^26^. However, the orbital rotation speed of a particle is relatively low^24,29,30^. Using the vortex beam with very high-order OAM, up to 86 Hz of orbital rotation has been recently reported^30^. And so far, it is still challenging to achieve high orbital rotation speed in an aqueous solution.

Furthermore, it is worth noting that in all the aforementioned studies, the diameter of orbital rotation is much larger than the diffraction limit, e.g., 4 μm in previous works^30^. The ability to rotate nanoparticles at subwavelength scale may reveal the light-matter interactions with nanometer spatial resolution and with subfemtosecond temporal resolution^31^, however, such task is difficult because it is hard to overcome the diffraction limit. Though superlenses^32^, metalenses^33^, and designed plasmonic structures^34^ have the potential to realize orbital rotation below the diffraction limit, these setups are either limited in the near field or in need of sophisticated nanofabrication, which limits their applications. Thus, another major challenge is to achieve beyond diffraction limit orbital rotation based on far-field technique.

In this study, we have achieved a stable orbital rotation at deep subwavelength scale, with record-high rotation frequency reaching 1013 Hz (at radius 213 nm) and record-small orbital radius of 71 nm (at frequency 521 Hz) under aqueous environment, based on the nonlinear optical effect of the particle in the far field. Unlike previous light-driven orbital rotations, such as those based on high-order Laguerre-Gaussian beams, we create this high-speed orbital rotation at subwavelength scale by merely utilizing a circularly polarized femtosecond Gaussian laser beam with a nonlinear particle. Detailed investigation of this system revealed that the nonlinearity of the particle material not only results in the rotation orbital, but also increases the conversion efficiency of photonic SAM to particle’s OAM, mediated by the surface plasmon resonance excited in the moving metallic particle.

## Results

### Experiment setup and results about orbital rotation of particles

**Fig. 1:**
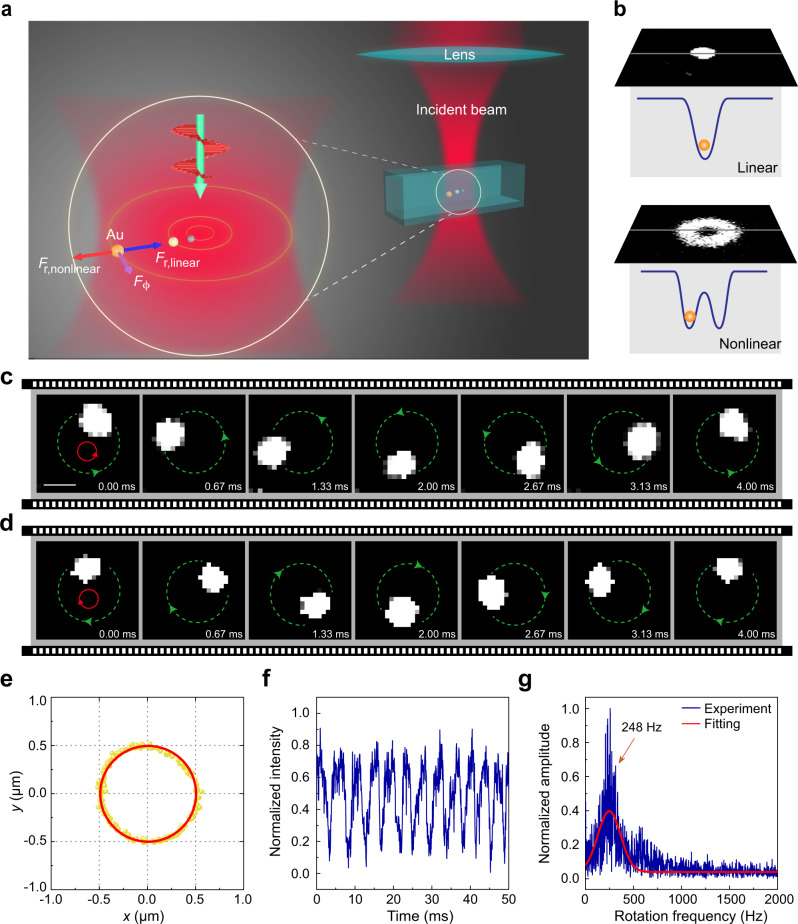
Illustration of the setup and typical experimental results for the movement trajectory of the trapped nonlinear particle. **a** Schematic of nonlinear nanoparticle (e.g., gold (Au), silver (Ag)) trapped by a circularly polarized Gaussian laser beam. **b** Image of the scattering light from the nonlinear gold nanoparticle (GNP) shows that the GNP was trapped in a ring shape potential well, when the incident beam was switched from continuous mode to femtosecond mode. **c**, **d** Successive frames of a movie recording, which show the rotation of a GNP trapped by a circularly polarized femtosecond laser with right handedness and left handedness, respectively. Images were taken by a CMOS camera at 1500 frames/s; green lines and arrows indicate the direction of GNP motion. Red arrows indicate the direction of handedness of circular polarization. The length of the white scale bars in all figures is 0.5 μm. **e** 2-D trajectory projection of an orbiting particle in the transverse plane recorded by front-side-view imaging system. **f** The intensity of the scattering light from the rotation particle is measured by the photomultiplier tube (PMT). **g** The Fourier transformation of the scattering intensity signal shows the rotation frequency. In Fig. 1b–g, the mean power *P* of trapping laser beam is about 630 mW, NA value of the trapping lens is 0.65.

The schematics of the nanoparticle circumgyration system is shown in Fig. 1a. Nonlinear spherical particles, gold nanoparticles (GNPs) with the radius of about 30 nm, are trapped by circularly polarized Gaussian laser beam with the wavelength of 840 nm (more in Methods and in Supplementary Note 1 of Supplementary Information). The GNPs were trapped in distilled water and illuminated by a continuous laser beam with green light (with wavelength of 532 nm), and thus could be observed by the scattered light or photoluminescence.

Without some particular preferred man-made orbit, particles are typically trapped in the center of the light beam in circularly polarized Gaussian beam. The SAM of the absorbed photons is transferred to the particles, resulting in spinning of the particles. To have the orbital rotation, we need to build an orbital for the particle. Compared to the previous methods that formed an orbit, such as schemes with nanofabrication or a laser beam with a particular phase structure, the method we proposed here is much simpler by applying a circularly polarized femtosecond Gaussian laser beam. Previous results have shown that a pair of point-like potential wells could be formed for nonlinear particles when the linearly-polarized femtosecond laser power exceeds a transition power^35–37^. By rotating the linear polarization of the laser, such potential-well pair could be rotated accordingly, thus resulting in subsequent circular movement of the trapped particle within the well. Therefore, it is intuitive to imagine that by using a circularly polarized beam, one could build an intrinsic ring-shape potential well, and the particle may be confined within while orbiting around the beam axis. In our experiment, the GNPs were irradiated with circularly polarized laser in the continuous-wave (CW) mode first. And there was only one trapping position located at the optical axis, as shown in the upper panel of Fig. 1b. When the laser was switched to femtosecond-pulse mode, a bright circle was observed around the beam center, as shown in lower panel of Fig. 1b. Thus, GNPs are indeed trapped in such a ring-shape potential well, as intuitively anticipated.

With a fast-speed camera, it was resolved that the bright circle was formed by the photoluminescence of the rapid rotating GNP around the beam axis in the experiment (the measured spectrum of the particle luminescence shown in Supplementary Note 1). The particle might rotate clockwise or counterclockwise, depending on the spin state of the incident light. Figure 1c and d showcase the typical results when a single GNP was trapped by a circularly polarized femtosecond laser with the right (also in Supplementary Movie 1) and left (also in Supplementary Movie 2) handedness, respectively. The bright area shows the position of the trapped GNP. The dashed green circle indicates the trajectory of the particle’s motion (i.e., the position of bright light circles mentioned above), and the arrow indicates the orbiting direction. The red arrow indicates the handedness of circular polarization. At the same power, the orbiting speed was identical for two kinds of handedness. The motion of the particle takes a three-dimensional (3D) trajectory in fact, though the motion distance along *z*-axis is short. With a side-view imaging system, the 3D trajectory was observed (in Supplementary Movie 3 and Supplementary Note 1). Along the *z*-axis, the scattering force shifts the equilibrium point from the beam center, and we estimated displacement shift about 1.3 micrometers. The 2-dimensional (2D) projection of the particle trajectory on the *xy*-plane during a few frames is shown in Fig. 1e as a typical example. We can obtain the orbital radius by fitting the projection data, and the orbital radius in Fig. 1e is about 491 nm (at mean trapping laser power of 630 mW). Using a PMT, we measured the orbital rotation frequency of the particle, as shown in Fig. 1f and g. The intensity of the scattering light from the rotation particle is measured by the PMT as shown in Fig. 1f. With the Fourier transformation, we can obtain the rotation frequency (248 Hz, laser power 630 mW) as shown in Fig. 1g. All the experimental data shown in Fig. 1 is measured by an objective with numerical aperture (NA) of 0.65 (Olympus LCPLN50XIR, 50X, NA = 0.65), and the laser power is the average power after the objective. It’s noted that multiple particles trapped simultaneously on the orbital have been also observed when high particle concentration was used (in Supplementary Movie 4), but we mainly focus on the single particle trapping and rotation in this work.

**Fig. 2:**
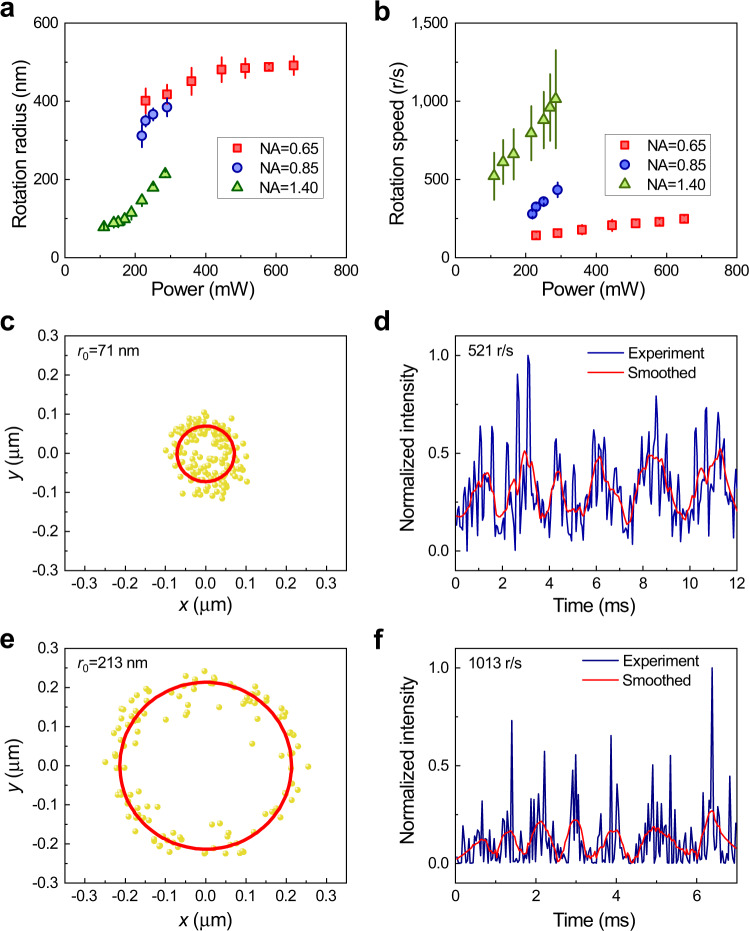
Experimental results for the high-frequency rotation at sub-diffraction scale. **a** Dependence of the orbital radius of the trapped particle (also the radius of the scattering light ring) on the mean laser beam power *P* with different NA lens, the rotation radii of particles under different NA are different. **b** Dependence of orbital speed of GNP on the mean power of trapped laser with different NA lens. With the control of the NA, we can control the rotation speed. In Fig. 2a, b, the standard deviation bars are included. **c** Two-dimensional trajectory projection on the *xy*-plane of an orbiting particle for the smallest orbital radius (about 71 nm) obtained in our experiments. **d** The intensity of the scattering light from the rotation particle when the radius of 71 nm is measured by the PMT. In Figs. (c, d), the mean power *P* of trapping laser beam is about 100 mW, NA = 1.40. **e**, **f** The same cases as in Fig. 2c, d, show the fastest orbital rotation (about 1013 Hz, the corresponding radius 213 nm) achieved in the experiments, where the mean power of the laser beam is about 285 mW, NA = 1.40.

The orbital radius and the orbital rotation speed of the GNPs are found to be dependent on the mean power of the femtosecond laser and NA of objective lens, as shown in Fig. 2a and b. Both the orbital radius and the speed increase as the laser power increasing. The influence of the NA value of objective lens on the rotation radius and the speed are also shown in Fig. 2a and b. When using an objective with NA = 1.40 (Olympus UPLSAPO100XO, 100X, NA = 1.40), the smallest orbital radius we achieved is about 71 nm (mean laser power of 100 mW) which is far beyond the diffraction limit (shown in Fig. 2c), and the corresponding orbital rotation frequency (Fig. 2d) is 521 Hz which is a relatively high orbital rotation speed in aqueous solution. The size of orbit is far beyond the diffraction limit, and we have determined it with the super-resolution imaging method based on single molecular localization^38^. And the highest frequency in our experiments is about 1013 Hz (orbital radius 213 nm) with the laser mean laser power 285 mW (as shown in Fig. 2e and f) which is the record-high orbital rotation frequency in an aqueous solution, to our best knowledge.

### Mechanism of ring-shape trapping and rotating

As the orbiting direction of spherical GNPs is totally dependent on the handedness of circular polarization of the incident Gaussian beam, and the orbiting speed is identical under the same power, the OAM of GNPs must be conversed from the SAM of incident Gaussian beam. It has been known that SAM to OAM conversion (STOC) could occur when a circularly polarized Gaussian beam is converged by a lens^29,39,40^, but the OAM cannot be transferred to GNPs under conventional optical trapping because the GNP is usually trapped in the beam axis. Trapping particle in the beam center results zero orbital rotation torque. In order to observe the conversed OAM, strategy such as an assisted orbit has been used, but the rotation speed was low^29^. The orbital rotation with superfast rotation speed in our experiment indicates there is a new STOC mechanism. The mechanism of trapping and accelerated rotating behind the experiment will be the key to understand and improve the rotation speed.

The formation of the ring shape potential well (also the rotation orbital) in circularly polarized Gaussian beam is easy to understand, as trap splitting in linear polarized Gaussian beam has been observed and explained considering the nonlinearity^35^. While the qualitative model in this Reference^35^ has explained the trapping behaviors very well, we give a quantitive model under the Rayleigh approximation here, which can predict the orbital radius based on the particle’s nonlinearity and the NA of lens (thus the field distribution of near the focus). Under the Rayleigh approximation, the radial trapping force near the trapping equilibrium point is ${F}_{{\rm{r}}}(r)=\frac{1}{4}{\varepsilon }_{{\rm{m}}}{\varepsilon }_{0}{\rm{Re}}\left(\alpha \right){\partial }_{r}{\left|{\bf{E}}(r)\right|}^{2}$ at the radial location *r* in the transverse plane, where $\alpha ={\alpha }_{0}/(1-\frac{i{k}_{{\rm{m}}}^{3}{\alpha }_{0}}{6\pi })$ is the effective polarizability of the particle and ${\bf{E}}(r)$ is the electric field at the particle’s location. For a Gaussian beam, the electric field is ${\bf{E}}(r)={{\bf{E}}}_{0}{e}^{-{{\rho }}^{2}}$ (where $\rho =r/w$ is the normalized radial location and $w$ is the half width of the beam waist). The static polarizability ${\alpha }_{0}=4\pi {a}^{3}\frac{{\varepsilon }_{{\rm{p}}}-{\varepsilon }_{{\rm{m}}}}{{\varepsilon }_{{\rm{p}}}+2{\varepsilon }_{{\rm{m}}}}$, where the radius of the spherical particle is $a$. The permittivity ${\varepsilon }_{{\rm{p}}}$ and ${\varepsilon }_{{\rm{m}}}$ are the relative permittivity of the nonlinear particle and the linear surrounding medium (relative to the vacuum permittivity ${\varepsilon }_{0}$), respectively. The wavevector in surrounding medium is ${k}_{{\rm{m}}}$. The trap potential well can be defined as then $U=-\int {F}_{{\rm{r}}}\left(r\right){dr}$. For particle with linear material, the potential well arrives at $U=-\frac{1}{4}{\varepsilon }_{{\rm{m}}}{\varepsilon }_{0}{\rm{Re}}\left(\alpha \right){\left|{\bf{E}}\right|}^{2}$ since $\alpha$ is independent the location *r*, and thus the particle is trapped at the beam center. However, for particle with nonlinearity, the potential well will be different. The relative permittivity of particles with nonlinearity reads

1 $${\varepsilon }_{{\rm{p}}}={\varepsilon }_{1}+{\chi }^{\left(3\right)}{E}^{2}.$$

Here, ${\varepsilon }_{1}={\varepsilon }_{1}^{{\prime} }+i{\varepsilon }_{1}^{{\prime} {\prime} }$ is the linear permittivity of the particle, ${\chi }^{\left(3\right)}$ is the third-order nonlinear susceptibility (also called third-order nonlinearity coefficient) and $E=\left|{\bf{E}}(r)\right|$ is the amplitude of the electric field. Now the permittivity ${\varepsilon }_{{\rm{p}}}$ and the polarizability $\alpha$ all depend on the particle’s location, the potential-well can be a ring shape and trap the particle away from the center at ${r}_{0}=\frac{w{\left({\rm{ln}}\frac{-{\chi }^{(3)}{E}_{0}^{2}}{{\varepsilon }_{1}^{{\prime} }+2{\varepsilon }_{{\rm{m}}}}\right)}^{\frac{1}{2}}}{\sqrt{2}}$ when $\frac{-{\chi }^{(3)}{E}_{0}^{2}}{{\varepsilon }_{1}^{{\prime} }+2{\varepsilon }_{{\rm{m}}}} > 1$ (see Supplementary Note 2), where ${E}_{0}$ is the maximum of field $E$. In other words, when trapped a GNP with nonlinearity, the trapping force on the particle in the radial direction can be written with two components as

2 $${F}_{{\rm{r}}}={F}_{{\rm{r}},{\rm{linear}}}+{F}_{{\rm{r}},{\rm{nonlinear}}}.$$

**Fig. 3:**
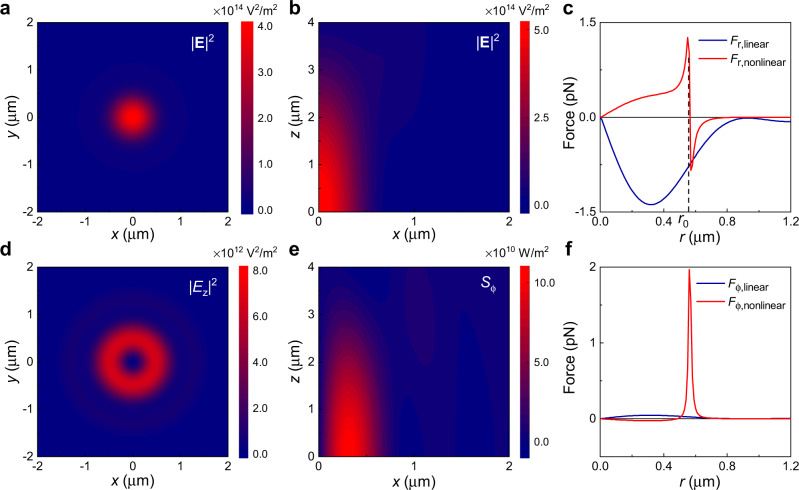
The focused incident field and the force acting upon a nonlinear particle. **a**, **b** The transverse (**a**) and longitude (**b**) electrical field |**E**^2^| distribution of the strongly focused circular polarized incident beam at $z=1.3$ μm. Incident beam with power $P=500{\ {\rm{mW}}}$ focused by a lens with NA = 0.65 in water. **c** A typical example of ${F}_{{\rm{r}}}$ with ${\chi }^{\left(3\right)}=2\times {10}^{-18}$ ${{\rm{m}}}^{2}/{{\rm{V}}}^{2}$, which has been decomposed into ${F}_{{\rm{r}},{\rm{linear}}}$ (red line) and ${F}_{{\rm{r}},{\rm{nonlinear}}}$ (blue line). **d** The ${\left|{E}_{{\rm{z}}}\right|}^{2}$ component distribution of the focused incident field, which will induce the azimuthal direction energy flux ${S}_{{\rm{\phi }}}$ as shown in Fig. 3**e**. **f** The azimuthal accelerating force ${F}_{{\rm{\phi }}}$ with its linear and nonlinear component caused by ${S}_{{\rm{\phi }}}$.

**Fig. 4:**
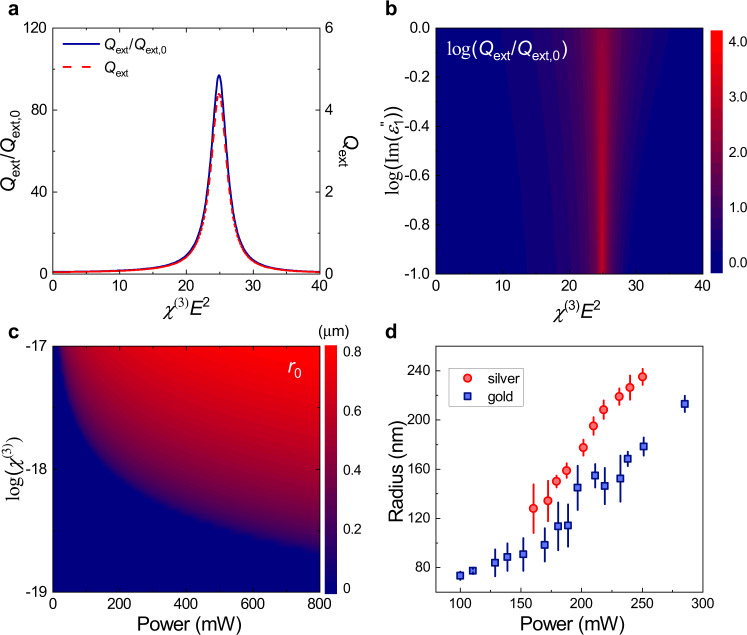
Analysis of extinction coefficient and orbital radius. **a** Extinction coefficient ${Q}_{{\rm{ext}}}$ (i.e., extinction cross-section normalized by the geometry cross-section $\pi {a}^{2}$) and its enhancement $\frac{{Q}_{{\rm{ext}}}}{{Q}_{{\rm{ext}},0}}$, where ${Q}_{{\rm{ext}},0}$ is the extinction coefficient without nonlinearity. **b** Extinction enhancement with different nonlinearities under various particle absorptions for GNPs. **c** Simulation result of the rotation radius ${r}_{0}$ under various incident power and particle nonlinearity for GNP with *a* = 30 nm. The light wavelength is 840 nm and NA = 0.65. **d** The experimental rotation radii of gold and silver particles. In Fig. 4d, the standard deviation bars are included. The different radii at the same power shows they have different nonlinearity.

The nonlinear part ${F}_{{\rm{r}},{\rm{non}}{\rm{linear}}}$ is only non-zero in the presence of particle’s nonlinearity. Since the third-order nonlinear susceptibility ${\chi }^{\left(3\right)}$ is very small, the nonlinearity shows impacts only in femtosecond pulse when the laser light intensity is strong enough. As an example, the linear part ${F}_{{\rm{r}},{\rm{linear}}}$ and nonlinear part ${F}_{{\rm{r}},{\rm{nonlinear}}}$ on gold nanoparticle are numerically calculated and shown in Fig. 3c for ${\chi }^{\left(3\right)}=2\times {10}^{-18}$ ${{\rm{m}}}^{2}/{{\rm{V}}}^{2}$. The linear permittivity of gold particle is ${\varepsilon }_{1}=-28.47+i1.361$ at wavelength 840 nm in our experiment. At the point $r={r}_{0}$, the two forces have opposite directions and are in balance, so the particle can be trapped. The trapping radius increases both with the beam power and nonlinearity ${\chi }^{\left(3\right)}$, as shown in Fig. 4c, which are consistent with our experimental data in Fig. 2a and Fig. 4d. The result suggests that the orbital radius can be controlled by laser power or material with different nonlinearity. In all, although the focused light field is in Gaussian mode approximately (Fig. 3a and b), the nonlinear effect of GNPs in femtosecond laser pulses generates an annular distribution of potential and confines the GNPs in the annular potential well.

As the SAM of circularly polarized Gaussian beam in our experiment can only spin the particle, it is the OAM converted from SAM that accelerates the rotation along the orbital. It is known that the SAM of the light can be converted to OAM after being focused^29,39,40^. Focused by the lens, the non-paraxial beam near the focus shows longitudinal electric field distribution and transverse energy flux around the beam center as shown in Fig. 3d and e. The transverse energy flux (corresponding to the OAM) pushes the particle and accelerates it to the maximum speed where the friction force of the environment equals the pushing force. Our experiment with observed superfast rotation frequency verifies the SAM to OAM conversion directly.

### Analysis of the improved rotation speed

The rotation speed along the orbital is much faster than expected. As a typical example in our experiment, the pushing force ${F}_{{\rm{\phi }}}$ is shown in Fig. 3f with its components (${F}_{{\rm{\phi }}}={F}_{{\rm{\phi }},{\rm{linear}}}+{F}_{{\rm{\phi }},{\rm{nonlinear}}}$) under the mean trapping power $P=500{\ }{\rm{mW}}$ (average ${E}_{0}^{2}\approx 4.15\times {10}^{14}\,{{\rm{V}}}^{2}/{{\rm{m}}}^{2}$), third-order nonlinear susceptibility ${\chi }^{\left(3\right)}=2\times {10}^{-18}\,{{\rm{m}}}^{2}/{{\rm{V}}}^{2}$ for the GNP (We choose this ${\chi }^{\left(3\right)}$ which predicts an orbital radius matching our experimental result. And it also falls within the experimental measured data^41^), and ${\rm{NA}}=0.65$. Without nonlinearity, the rotation speed is estimated to be 41.9 r/s at power 500 mW, much lower than the 220 r/s observed. The pushing (azimuthal) force can be calculated with approximate formula as ${F}_{{\rm{\phi }}}=\frac{{n}_{{\rm{m}}}{C}_{{\rm{ext}}}{S}_{{\rm{\phi }}}}{c}$, where the energy flux in the azimuthal direction is ${S}_{{\rm{\phi }}}$ and the extinction cross-section of the particle is ${C}_{{\rm{ext}}}$. The extinction coefficient ${Q}_{{\rm{ext}}}$ is defined as the extinction cross-section normalized by the geometry cross-section $\pi {a}^{2}$. For the nonlinear particle located in the electromagnetic field, the extinction coefficient is (see Supplementary Note 3)

3 $${Q}_{{\rm{ext}}}=4{k}_{{\rm{m}}}a{\rm{Im}}\left[\frac{{\varepsilon }_{{\rm{p}}}-{\varepsilon }_{{\rm{m}}}}{{\varepsilon }_{{\rm{p}}}+{2\varepsilon }_{{\rm{m}}}-\frac{2i}{3}{\left({k}_{{\rm{m}}}a\right)}^{3}\left({\varepsilon }_{{\rm{p}}}-{\varepsilon }_{{\rm{m}}}\right)}\right].$$

Extinction coefficient ${Q}_{{\rm{ext}}}$ and its enhancement $\frac{{Q}_{{\rm{ext}}}}{{Q}_{{\rm{ext}},0}}$, where ${Q}_{{\rm{ext}},0}$ is the extinction coefficient without nonlinearity, are calculated with different ${\chi }^{\left(3\right)}{E}^{2}$ and shown in Fig. 4a. It shows that the extinction coefficient has a resonant peak when ${\varepsilon }_{1}^{{\prime} }+{\chi }^{\left(3\right)}{E}^{2}+2{\varepsilon }_{{\rm{m}}}=0.$ And this is also the condition that the particle can be trapped in the orbital with orbital radius ${r}_{0}$. Moreover, nonlinearity induced permittivity change ${\chi }^{\left(3\right)}{E}^{2}$ can increase ${Q}_{{\rm{ext}}}$ dramatically with even several orders of magnitude. Thus, we have found the increased speed is due to the enhanced extinction cross-section of the particle. Considering the nonlinearity, the rotation speed is estimated about 4067 r/s at power 500 mW in our experiment, about two orders higher. There are the additional deduction factors in experiment (such as the bubbling around the particle, the none-perfect orbital, etc.), the lower experimental observed speed 220 r/s is reasonable. The simulation shows that the azimuthal force greatly enhanced because of the nonlinear effect (as shown in Fig. 3f), which greatly enhanced the torque exerted on the GNPs and further results in the superfast rotation. In total, the conversion efficiency of the photonic SAM to the particle’s OAM has been improved by the particle nonlinearity.

In addition, we analyzed the extinction enhancement theoretically. It is noted in Eq. (3) that the ${Q}_{{\rm{ext}}}$ has a resonant point at ${\varepsilon }_{1}^{{\prime}}+{\chi }^{\left(3\right)}{E}^{2}+{2\varepsilon }_{{\rm{m}}}=0$ (more in Supplementary Note 3). This is the point where the surface plasmonic resonance of a metallic particle can be excited^42^. The maximum of ${Q}_{{\rm{ext}}}$ is proportional to $\frac{1}{{\varepsilon }_{1}^{{\prime} {\prime} }}$ when the absorption is strong, which means the extinction cross-section, thus the rotation speed of a particle, can be improved if we engineer the particle material to be less absorptive (i.e., smaller ${\varepsilon }_{1}^{{\prime} {\prime} }$). Of course, when the absorption is minor or comparable relative to the scattering, especially when the system is near the resonant point, the radiative part cannot be eliminated. At the resonant point, it is shown that surface plasmon resonance happens, and the ${Q}_{{\rm{ext}}}$ will be limited by the scattering with ${Q}_{{{\max }}}=6\pi /{\left({k}_{{\rm{m}}}a\right)}^{2}$ (more in Supplementary Note 3).

## Discussion

The rotations with such high frequency and subwavelength dimensional orbital radius achieved in our experiments are very suggestive of micromachines or applications in nano-rheology. The orbital radius and the rotation speed depend on the laser power and nonlinearity *χ*^(3)^ as we discussed above, so the rotation can be designed by adjusting laser power or choosing different materials of the particle. Besides, as the distribution of light intensity, as while as *z*-component of field after focusing is highly dependent on the NA of objective lens, the rotating rate and the radius of orbit can also be adjusted by the value of NA. By adjusting NA, power and material, the desired radius and speed can be obtained, as demonstrated in our experiments (Fig. 2a, b and Fig. 4d).

In conclusion, we have experimentally demonstrated a nanoscale circumgyration system with record-high orbital rotation frequency 1013 Hz of nanoparticles in water (corresponding orbiting radius 213 nm) and record-small orbiting radius of 71 nm (corresponding speed 521 Hz). The trap splitting induced by particle’s nonlinearity generates an annular shape of the potential-well in femtosecond laser pulses of fundamental Gaussian mode, without resorting to incident vortex beams. As the orbit is derived from the split of a Gaussian beam based on nonlinear interaction, the size of orbit will overcome the diffraction limit, much less than any optical vortex. Such circumgyration phenomenon reveals a new mechanism and improved efficiency of angular momentum conversion to particles assisted by surface plasmon resonance, besides direct verifying the STOC during the beam focusing. The surface plasmon resonance enhanced extinction cross-section plays important roles in the circumgyration, which overcomes the increased resistance induced by cavitation bubbling and orbital perturbation. Our work not only provides fundamental insights into the interaction of light and matter, but also suggests potential applications in various fields, such as optical micromachines, nano-rheology, laser microfabrication, and femtosecond laser nano-surgery of cells and tissues.

## Methods

### Experiment setup

A single beam laser trapping system was constructed on an optical vibration isolation table, as shown in Supplementary Fig. 1. A Ti:sapphire laser (Mai Tai,Spectra-Physics, USA) was used for the optical trapping, and its output can be readily switched between CW and femtosecond-pulse modes. In both operation modes, the laser provides a Gaussian beam in TEM00 transverse mode. The wavelength of the Ti:sapphire laser was set to 840 nm. The pulse width and repetition rate of the optical pulses are 70 fs and 80 MHz, respectively, and the maximal output of mean power is approximately 2.5 W at 840 nm. The laser beam was expanded by a telescope (L1 and L2), and the linear polarization after beam expansion was confirmed by a near-infrared polarizer. A combination of half-wave plate (HWP) and polarizing beam splitter (PBS) adjust the laser power. The power was monitored by a power meter (PM, S425C, Thorlabs, USA). The circular polarization could be adjusted by a quarter-wave plate (QWP) before introducing the beam to the objective lens (OL1). The OL1 was used both for optical trapping and front view observation. It was installed with three types of objective lens (LCPLN50XIR, 50X, NA=0.65; LCPLN100XIR, 100X, NA=0.85; and UPLSAPO100XO, 100X, NA=1.40, Olympus, Japan). Another objective lens (OL2, M Plan Apo SL, 50X, NA 0.42, Mitutoyo, Japan) was used for side-view observation.

### Materials and measurement methods

A square glass tube was used as a sample cell as shown in Supplementary Fig. 1. The sample gold nanoparticles (GNPs) with a diameter of 60 nm were purchased from BBI International, USA and the SEM we got is shown in Supplementary Fig. 2a. The GNPs were suspended in distilled water and the concentration can be adjusted according to the experimental requirements. As shown in Supplementary Fig. 1, the particles were illuminated by a 532-nm laser via an optical fiber inserted into the top of the tube. When the angle between the illuminating light and tube wall is less than the critical angle, the light will be totally internally reflected by the outer surface of the glass tube. This part of light was confined inside the tube and propagated along the tube, while the other part of the illuminating light escaped at the top of the tube. The GNPs inside the tube were illuminated by the light, and the light scattered from the GNPs could pass through the tube walls and were observed by the imaging system. For both OL1, OL2 and OL3, these configurations realized dark-field illumination conditions. The front-view image of trapped particles was observed by a high-speed camera (CCD1, sCMOS, PCO.EDGE 5.5, PCO-Tech Inc., Germany) through OL1. The orbital rotation frequency of the particle was measured by a photomultiplier tubes (PMT, PMM02, Thorlabs, USA). The maximal sampling rate of the PMT is 20 kHz. An aperture (A1, VA100/M, Thorlabs, USA) between the OL1 and the PMT was used to block part of the particle trajectory. The side-view image and front-view image of trapped particles was observed synchronously by another high-speed camera (CCD2, sCMOS, PCO.EDGE 5.5, PCO-Tech Inc., Germany) through OL2 and OL3. The maximal sampling rate of the sCMOS camera is 1500 frames/s, so the dynamics of trapped particles could be resolved. Then, the three-dimensional distribution of trapped particles can be determined (as shown in Supplementary Fig. 2b as an example). A commercial spectrometer (QE Pro-FL, Ocean Optics, USA) was combined with the trapping system, so that the luminescence spectra emitted from the trapped gold particles could be measured.

## Supplementary information

Supplementary Information

Description of Additional Supplementary Files

Supplementary Movie 1

Supplementary Movie 2

Supplementary Movie 3

Supplementary Movie 4

## Data Availability

All datasets generated and/or analyzed during the current study are available from the corresponding author on reasonable request.
