# Supplementary material for: Nonlinearity-induced nanoparticle circumgyration at sub-diffraction scale
Source: Nat Commun. 2021 Jun 17;12:3722. doi: 10.1038/s41467-021-24100-0 (PMC8211862; doi:10.1038/s41467-021-24100-0)
Supplement: Supplementary file 1 — Supplementary Information [file 41467_2021_24100_MOESM1_ESM.pdf]

Supplementary Information to: Nonlinearity-induced Nanoparticle Circumgyration at Sub-diffraction Scale

April 22, 2021

Yaqiang Qin [1,2,3#], Lei-Ming Zhou [4,5#], Lu Huang [1,2,3#], Yunfeng Jin [1#], Hao Shi [1], Shali Shi [6], Honglian Guo [6], Liantuan Xiao [7], Yuanjie Yang [8*], Cheng-Wei Qiu [4*], Yuqiang Jiang [1,2,3,7*]

1 State Key Laboratory of Molecular Developmental Biology, Institute of Genetics and Developmental Biology, Chinese Academy of Sciences, Beijing, 100101, China

2 Innovation Academy for Seed Design, Chinese Academy of Sciences, Beijing, 100101, China

3 University of Chinese Academy of Sciences, Beijing, 100049, China

4 Department of Electrical and Computer Engineering, National University of Singapore, Singapore 117583, Singapore

5 Department of Optical Science and Engineering, Hefei University of Technology, Hefei, Anhui 230009, China

6 College of Science, Minzu University of China, Beijing, 100081, China

7 State Key Laboratory of Quantum Optics and Quantum Optics Devices, Collaborative Innovation Center of Extreme Optics, Institute of Laser Spectroscopy, Shanxi University, Taiyuan, 030006, China

8 School of Physics, University of Electronic Science and Technology of China, Chengdu 610054, China

#These authors have same contribution to the work;

* Correspondence: dr.yang2003@uestc.edu.cn;chengwei.qiu@nus.edu.sg; yqjiang@genetics.ac.cn.

Supplementary Note 1: Experiment setup and materials

The experiment setup is shown in Supplementary Figure 1 and the SEM images of the gold nanoparticles used in our experiment are shown in Supplementary Figure 2.

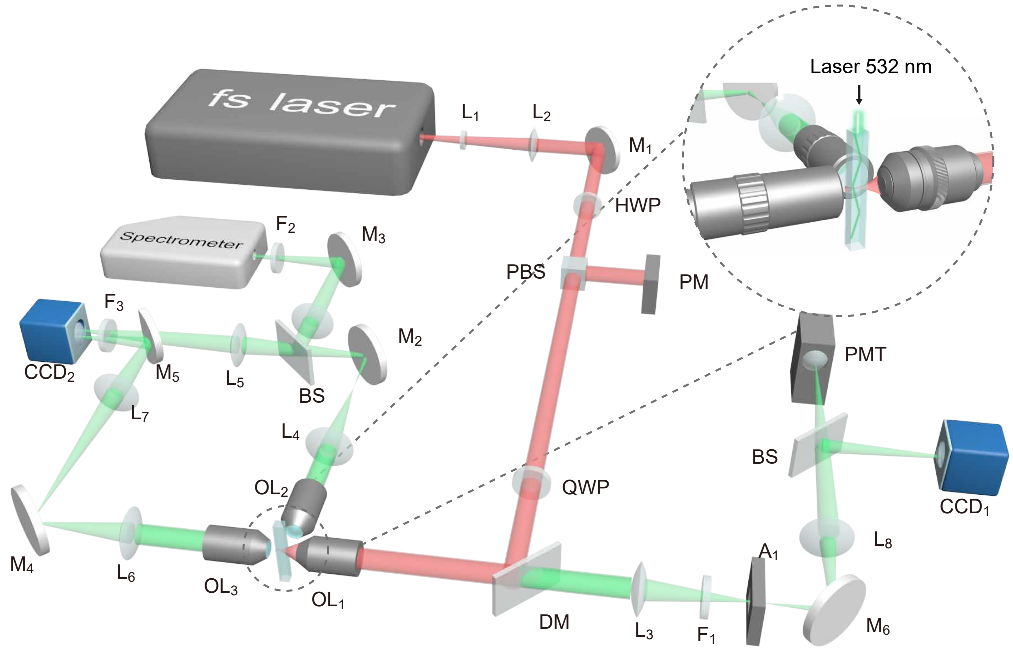

Supplementary Figure 1. Schematic of the experiment setup. More descriptions can be found in the Methods section of the main text.

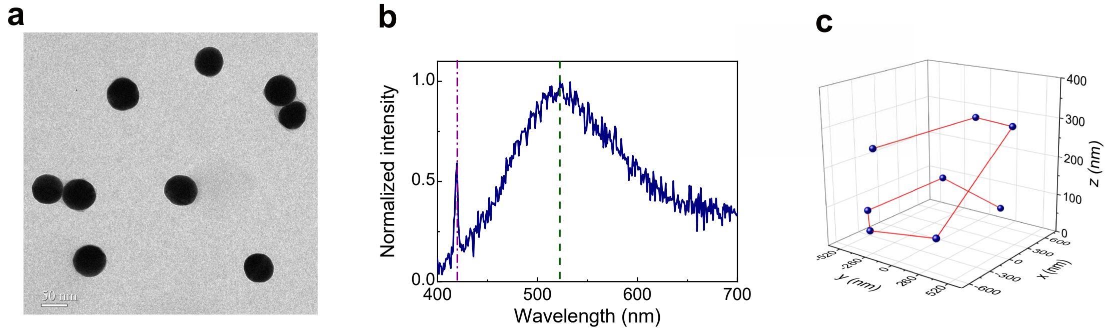

Supplementary Figure 2. (a) SEM image of the gold particle in our experiment. (b) The spectrum of the luminescence from the trapped particle. (c) 3-D trajectory of the trapped particle shows that the particle's motion is mainly in the transverse plane.

Supplementary Note 2: Simulation of the optical force and the trapping behavior of nonlinear particle

During the experiment of trapping metallic particle with ultrashort laser pulses, the particle can be trapped at a location away from the beam center as shown in the main text. While the numerical result of force has been shown in the main text, here we would like to describe the investigation model and method used.

The trapping beam has been focused by high numerical aperture (NA) lens. After strong focusing, the beam is no more paraxial Gaussian beam. The focused field has been calculated with vector Debye integral theory [1]. After focusing, the field at point (ρ,φ,z) near the focus is [1]:

$$\mathbf{E}(\rho,\varphi,z)=-\frac{ikfe^{-ikf}}{2\pi}\int_0^{\theta_{max}}\int_0^{2\pi}\mathbf{E}_\infty(\theta,\phi)e^{ik\rho\sin\theta\cos(\phi-\varphi)}e^{ikz\cos\theta}\sin\theta\mathrm{d}\theta\mathrm{d}\phi. \tag{S1}$$

Similarly, we can get the field $\mathbf{H}$. The energy flux density $\bar{\boldsymbol{S}}=\mathrm{Re}(\mathbf{E}\times\mathbf{H}^*)/2$ then can be calculated, which has been shown in the main text.

With the calculated electromagnetic field, we calculate the optical force exerted on the metallic particle under Rayleigh approximation. In our experiment, the particle radius is about 30 nm, which falls into the regime of this approximation (i.e., $k_\mathrm{m}a\ll 1$). The effective polarizability of the particle thus is

$$\alpha=\alpha_0/(1-ik_\mathrm{m}^3\alpha_0/(6\pi)), \tag{S2}$$

where $\alpha_0=4\pi a^3\frac{\varepsilon_\mathrm{p}-\varepsilon_\mathrm{m}}{\varepsilon_\mathrm{p}+2\varepsilon_\mathrm{m}}$ is the static effective polarizability [2], the radius of the spherical particle is a, the light wavelength in vacuum is λ_0, the refractive index of the surrounding medium is n_m, and $k_\mathrm{m}=2\pi n_\mathrm{m}/\lambda_0$. The relative permittivity of the particle and surrounding medium is ε_p and ε_m respectively, where $\varepsilon_\mathrm{m}=n_\mathrm{m}^2$ and $\varepsilon_\mathrm{p}=n_\mathrm{p}^2$ (the refractive index of the particle is n_p). The gradient force exerted on the particle thus reads

$$\mathbf{F}_\text{grad}=\frac{1}{4}\varepsilon_\mathrm{m}\varepsilon_0\mathrm{Re}(\alpha)\nabla|\mathbf{E}|^2. \tag{S3}$$

And the scattering force is

$$\mathbf{F}_\text{scat}=\frac{n_\mathrm{m}\sigma_\text{ext}\mathbf{S}}{c}, \tag{S4}$$

where σ_ext is the extinction cross-section of the particle and c is the velocity of light in vacuum.

For dielectric particles with positive permittivity,the particle can either be trapped or pushed away along the z axis, depending on the competition of the gradient force and the scattering force; and in the transverse plane, the particle can be trapped in the center of the intensity spot. For metallic particles, the permittivity

is negative for a large range of wavelength, and the behaviors of force can be different. Taking the gradient force exerted on the particle in the transverse plane of a Gaussian beam as an example, the gradient force can opposite to the gradient of the electromagnetic filed intensity. Without loss of validity, we show this point using the static effective polarizability $\alpha_0 = 4\pi a^3(\varepsilon_\mathrm{p} - \varepsilon_\mathrm{m})/(\varepsilon_\mathrm{p} + 2\varepsilon_\mathrm{m})$ instead of α first. The permittivity of metallic particle is ε_p and the permittivity of surrounding medium is ε_m. When $\varepsilon_\mathrm{p} \in [-\infty, -2\varepsilon_\mathrm{m})$, $\alpha > 0$, the gradient force points toward the beam center and can trap the particles; When $\varepsilon_\mathrm{p} \in (-2\varepsilon_\mathrm{m}, 0)$, $\alpha < 0$, the gradient force points outwards and pushes the particle away from the beam center. In our case of nonlinear particle in Gaussian beam, $\varepsilon_\mathrm{p} = \varepsilon_\mathrm{p}(E(r)) = \varepsilon_\mathrm{p}(r)$. When the particle location $r < r_0$ (where r_0 is the location satisfying $\varepsilon_\mathrm{p}(r_0) + 2\varepsilon_\mathrm{m} = 0$), the particle is pushed outwards; When $r > r_0$, the particle is pulled toward the beam center. Thus, it'spossible to trap the particle near $r = r_0$.

Considering particle material with absorption and third order nonlinearity $\chi^{(3)}$, we denote the permittivity of the particle in the electrical field E as

$$\varepsilon_\mathrm{p} = \varepsilon_1^{'} + i\varepsilon_1^{''} + \chi^{(3)}E^2. \tag{S5}$$

The real and imaginary part of the linear permittivity are denoted $\varepsilon_1^{'}$ and $\varepsilon_1^{''}$ respectively. Suppose the incident beam is a Gaussian beam, the electrical field distribution in its transverse plane is

$$E(\rho, z_0) = |\mathbf{E}| = E_0 e^{-\rho^2}, \tag{S6}$$

where E_0 is the maximum electric field amplitude, $\rho = r/w$ is the normalized displacement and w is the half width of the beam profile. For the homogeneous and isotropic gold metallic particle here, the permittivity can then be expressed as

$$\varepsilon_\mathrm{p} = \varepsilon_1^{'} + i\varepsilon_1^{''} + \Delta\varepsilon_3 e^{-2\rho^2}, \tag{S7}$$

where $\Delta\varepsilon_3 = \chi^{(3)}E_0^2$. On the transverse plane of light propagation, with the gradient force calculated, the potential function can be obtained by $U(\rho) = -\int F_\mathrm{grad}(\rho)\mathrm{d}\rho$. Suppose $\alpha \approx \alpha_0$ and the dependence of permittivity $\varepsilon_\mathrm{p}(\rho)$ on location ρ is much less than that of the electrical field $E(\rho)$, we get an approximate the potential function as $U_0 = -\frac{1}{4}\varepsilon_\mathrm{m}\varepsilon_0 \mathrm{Re}(\alpha_0)|\mathbf{E}|^2$. Normalized by a coefficient $U_c = \pi a^3 \varepsilon_\mathrm{m}\varepsilon_0 E_0^2$, we get an approximated potential

$$U_\mathrm{approx} = -\mathrm{Re}(\frac{\varepsilon_\mathrm{p} - \varepsilon_\mathrm{m}}{\varepsilon_\mathrm{p} + 2\varepsilon_\mathrm{m}})e^{-2\rho^2}. \tag{S8}$$

For particle material without absorption, the permittivity is supposed to be a real number, i.e., $\varepsilon_1^{''} = 0$.

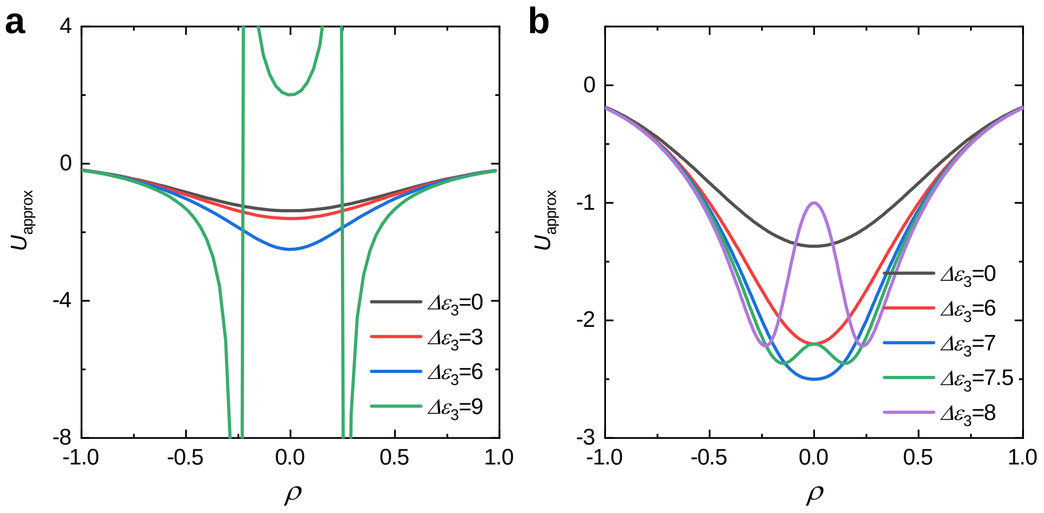

Supplementary Figure 3. (a) The normalized approximate potential function U_{approx} with different $\Delta\varepsilon_3$ when particle's linear permittivity $\varepsilon_1^{'} = -10$ and $\varepsilon_1^{''} = 0$ (no absorption). (b) The normalized potential function U_{approx} with the different nonlinearity $\Delta\varepsilon_3$ when $\varepsilon_1^{'} = -10$ and $\varepsilon_1^{''} = 1.0$. In all cases, $\varepsilon_{\mathrm{m}} = 1$.

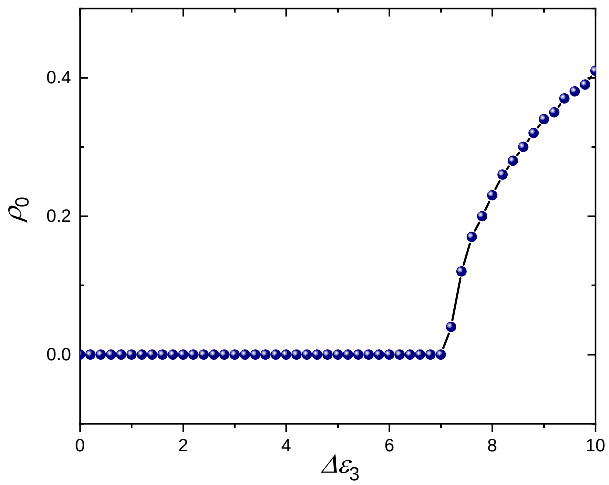

Supplementary Figure 4. The location of the trap potential minimum ρ_0 for various $\Delta\varepsilon_3$ when $\varepsilon_1^{'} = -10$, $\varepsilon_1^{''} = 1.0$ and $\varepsilon_{\mathrm{m}} = 1$.

The normalized potential function is

$$U_{\text{approx}} = -\frac{\varepsilon_1^{'} + \triangle\varepsilon_3 e^{-2\rho^2} - \varepsilon_{\text{m}}}{\varepsilon_1^{'} + \triangle\varepsilon_3 e^{-2\rho^2} + 2\varepsilon_{\text{m}}} e^{-2\rho^2}. \tag{S9}$$

The shape of U_{approx} is shown in Supplementary Figure 3(a). Suppose that the magnitude of nonlinearity-induced permittivity change is smaller than the original linear permittivity [3] (i.e., $aI_0 < 1$ in Ref. [3], or $\varepsilon_1^{'} + \Delta\varepsilon_3 < 0$ in our model), we have plotted cases when $\Delta\varepsilon_3 < 10$. Other parameters are shown in the figure caption. The normalized potential function splits when $\Delta\varepsilon_3$ is large enough. It's noted that the potential has no minimum point in fact, but a singular point when the denominator is zero, i.e., $\varepsilon_1^{'} + \Delta\varepsilon_3 e^{-2\rho^2} + 2\varepsilon_{\text{m}} = 0$. Then we get the singular or transition point as

$$\rho_0 = (\ln(\frac{-\chi^{(3)} E_0^2}{\varepsilon_1^{'} + 2\varepsilon_{\text{m}}})/2)^{\frac{1}{2}}. \tag{S10}$$

It can be seen that the potential well splits when $\frac{-\chi^{(3)} E_0^2}{\varepsilon_1^{'} + 2\varepsilon_{\text{m}}} > 1$ and there is a potential minimum at $r_0 = \rho_0 w$.

For particle material in experiments with both nonlinearity and absorption, we have

$$U_{\text{approx}} = -\text{Re}(\frac{\varepsilon_1^{'} + i\varepsilon_1^{''} + \Delta\varepsilon_3 e^{-2\rho^2} - \varepsilon_{\text{m}}}{\varepsilon_1^{'} + i\varepsilon_1^{''} + \Delta\varepsilon_3 e^{-2\rho^2} + 2\varepsilon_{\text{m}}}) e^{-2\rho^2} \tag{S11}$$

$$= -\text{Re}(\frac{(\varepsilon_1^{'} + \Delta\varepsilon_3 e^{-2\rho^2} - \varepsilon_{\text{m}})(\varepsilon_1^{'} + \Delta\varepsilon_3 e^{-2\rho^2} + 2\varepsilon_{\text{m}}) + \varepsilon_1^{''2} + i3\varepsilon_{\text{m}}\varepsilon_1^{''}}{(\varepsilon_1^{'} + \Delta\varepsilon_3 e^{-2\rho^2} + 2\varepsilon_{\text{m}})^2 + \varepsilon_1^{''2}}) e^{-2\rho^2}. \tag{S12}$$

When the material has very minor absorption (i.e., $\varepsilon_1^{''} \ll |\varepsilon_1^{'}|$), omitting the second and higher order terms of $\varepsilon_1^{''}$ in Eq. (S12), we again arrive at Eq. (S9). The cases for various $\Delta\varepsilon_3$ are shown in Supplementary Figure 3(b) when $\varepsilon_1^{'} = -10$ and $\varepsilon_1^{''} = 1.0$. It can be seen that the trapping radius ρ_0 increases with the $\Delta\varepsilon_3$. The dependence of ρ_0 on $\Delta\varepsilon_3$ is shown in Supplementary Figure 4. It's remarked that the potential U_{approx} is an approximation, supposing that the dependence of effective permittivity $\varepsilon_{\text{p}}(\rho)$ on location ρ is much less than that of the electrical field $E(\rho)$ [4]. But this appropriate model can help us predict the location of potential minimum.

When $\varepsilon_1^{'} + \Delta\varepsilon_3 = -2\varepsilon_{\text{m}}$, it is the plasmonic resonance point [5]. At the resonance point, the scattering is strongly enhanced and the extinction is improved as we will discuss it in the below, which dramatically improve the orbital angular momentum conversion to the particle from the spin angular momentum of incident light. Investigation of the scattering near the resonance with other numerical methods proved that the dipole approximation used here still works well [5].

Supplementary Note 3: Accelerated rotation speed of the trapped particle by nonlinearity

Rotation speed of the trapped particle. The pushing force F_ϕ induced by the transverse angular energy flux (accompanying the orbital angular momentum (OAM) conversed from spin angular momentum (SAM)) accelerates the particle until it equals the viscous resistance force f_v caused by the particle's motion in water. Here, $f_\nu = 6\pi\eta va$ is given by the Stokes equation, where η is dynamical viscosity of water ($\eta = 282.1$Pa . s at 100°C [6]), a is the radius of the GNP, and v the speed of the GNP. The pushing force can be calculated with approximate formula as $F_\phi = (n_\text{m} C_\text{ext} S_\phi)/c$, where the energy flux is S_ϕ and the extinction cross-section of the particle is C_ext. The optical velocity constant is c and the refractive index of medium water is $n_\text{m} = 1.333$. Under the Rayleigh approximation, supposing that the GNP is linear with permittivity $\varepsilon_\text{p} = -28.47 + 1.361i$ at light wavelength 840 nm [7], the linear extinction cross-section $C_\text{ext} = 0.0454\pi a^2$. For incident beam with mean power $P = 500$ mW, the mean energy flux is $S_\phi = 4.16 \times 10^{10}\text{W/m}^2$ on the theoretical orbital with radius $r_0 = 564$ nm (numerically calculated in Fig. 3c). The rotation speed is estimated to be 41.9 r/s at most, much lower than experimental observed speed. In our experiment, the particle with power $P = 500$ mW rotated at about 220 r/s.

Besides, the heating effect named cavitation indicates that the gold particles may be surrounded by bubbles in water, which will slow the rotation speed. Cavitation will occur when GNPs are irradiated and trapped by intense ultrashort laser pulses [8]. Considering the conditions of the femtosecond laser that we used for optical trapping, the power density of the laser pulse is estimated to be above the threshold of cavitation (approximately 200mJ/cm^2) [9]. Considering our experimental conditions, the radius of the bubble is estimated to be 50 nm. Hence, the radius of particles $a = 30$ nm in motion equation should be replaced by the radius of the bubble $r_b = 50$ nm in the above analysis. Then the orbiting speed will be much lower. It's shown below that it is the nonlinearity that increases the rotation speed.

Extinction coefficient enhancement. Under the Rayleigh approximation, the extinction cross-section is

$$C_\text{ext} = k_\text{m}\text{Im}(\alpha). \tag{S13}$$

It's noted that we use the radiation corrected effective polarizability α here and $\alpha = \alpha_0/(1 - ik_\text{m}^3\alpha_0/6\pi)$. Normalized by the geometrical cross-section $C_\text{geo} = \pi a^2$ of the spherical particle with radius a, the extinction coefficient is

$$Q_\text{ext} = \frac{C_\text{ext}}{C_\text{geo}} = 4k_\text{m}a\text{Im}[\frac{\varepsilon_\text{p} - \varepsilon_\text{m}}{\varepsilon_\text{p} + 2\varepsilon_\text{m} - \frac{2}{3}i(k_\text{m}a)^3(\varepsilon_\text{p} - \varepsilon_\text{m})}]. \tag{S14}$$

Substituting the nonlinear $\varepsilon_{\text{p}} = \varepsilon_1^{'} + i\varepsilon_1^{''} + \triangle\varepsilon_3 e^{-2\rho^2}$, we get

$$Q_{\text{ext}} = 4k_m a \text{Im}[\frac{\varepsilon_1^{'} + i\varepsilon_1^{''} + \triangle\varepsilon_3 e^{-2\rho^2} - \varepsilon_{\text{m}}}{\varepsilon_1^{'} + i\varepsilon_1^{''} + \triangle\varepsilon_3 e^{-2\rho^2} + 2\varepsilon_{\text{m}} - \frac{2}{3}i(k_m a)^3(\varepsilon_1^{'} + i\varepsilon_1^{''} + \triangle\varepsilon_3 e^{-2\rho^2} - \varepsilon_{\text{m}})}]. \tag{S15}$$

When the absorption is strong, we eliminate the radiation correction term (i.e., the last term of the denominator in Eq. (S14)) and have

$$\begin{aligned} Q_{\text{ext,abs}} &\approx 4k_m a \text{Im}[\frac{\varepsilon_1^{'} + i\varepsilon_1^{''} + \triangle\varepsilon_3 e^{-2\rho^2} - \varepsilon_{\text{m}}}{\varepsilon_1^{'} + i\varepsilon_1^{''} + \triangle\varepsilon_3 e^{-2\rho^2} + 2\varepsilon_{\text{m}}}] \\ &= \frac{12k_m a\varepsilon_1^{''}\varepsilon_{\text{m}}}{(\varepsilon_1^{'} + \triangle\varepsilon_3 e^{-2\rho^2} + 2\varepsilon_{\text{m}})^2 + \varepsilon_1^{''2}}. \end{aligned} \tag{S16}$$

The $Q_{\text{ext,abs}}$ has a resonant peak with $\triangle\varepsilon_3 e^{-2\rho^2}$, which sits at the same radial location as the minimum of the split potential-trap does. The reduction of the absorption will increase the $Q_{\text{ext,abs}}$.

However, when the absorption is much smaller than the radiation correction term, or the system is near the resonant point (i.e., $\varepsilon_1^{'} + \triangle\varepsilon_3 e^{-2\rho^2} + 2\varepsilon_{\text{m}} = 0$), the radiation (i.e., light scattering of the particle) is strong. Now we eliminate the absorption and have

$$Q_{\text{ext,sca}} \approx 4k_{\text{m}} a \text{Im}[\frac{\varepsilon_1^{'} + \triangle\varepsilon_3 e^{-2\rho^2} - \varepsilon_{\text{m}}}{\varepsilon_1^{'} + \triangle\varepsilon_3 e^{-2\rho^2} + 2\varepsilon_{\text{m}} - \frac{2}{3}i(k_{\text{m}} a)^3(\varepsilon_1^{'} + \triangle\varepsilon_3 e^{-2\rho^2} - \varepsilon_{\text{m}})}]. \tag{S17}$$

When there is no particle absorption, and $\varepsilon_1^{'} + \triangle\varepsilon_3 e^{-2\rho^2} + 2\varepsilon_{\text{m}} = 0$, we have the maximum of Q_{ext} as $Q_{\text{max}} = 6/(k_{\text{m}} a)^2$. This is the maximum of the enhanced extinction coefficient. For $a = 30$ nm in our experiment, Q_{ext} is estimated to be 4.40, i.e., which has been improved about 100 times compared to the linear case. The theory here thus matches the experiment result (the resident error may be induced by the bubble size estimation, the none-perfect orbital, etc.). The nonlinearity induced increase of C_{ext} also results in the increase of the OAM of particle moving along its orbital. Thus, in total, the conversion efficiency of the optical SAM to particle's OAM has been increased too.

Supplementary Note 4: Tune/control the rotation orbital and speed

The orbital images of different power are shown Supplementary Figure 5. We got the record of minimum orbital radius of 71 nm when the laser power is 100 mW for gold particle. As we have discussed, the orbital radius of the particle depends on both the laser power and the particle's nonlinearity. We also tried the silver particle . The potential trap shows the same behaviors as that of the gold particle does. The trapping radius is shown in Fig. 4d of the main text. At the same power, the silver particles take larger orbital radii and thus

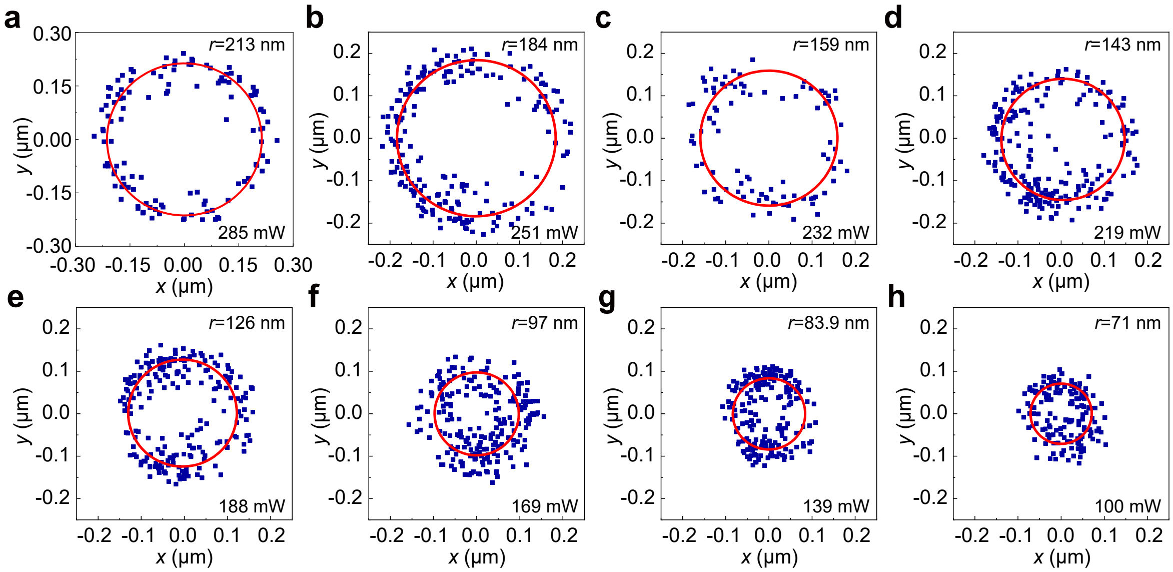

Supplementary Figure 5. Various trapping orbitals under different power for gold particles .

imply a higher third order nonlinearity coefficient.

Supplementary References

[1] Lukas Novotny and Bert Hecht. *Principles of nano-optics*. Cambridge University Press, 2012.

[2] Karel Svoboda and Steven M. Block. Optical trapping of metallic rayleigh particles. *Opt. Lett.*, 19(13):930–932, 1994.

[3] Yuqiang Jiang, Tetsuya Narushima, and Hiromi Okamoto. Nonlinear optical effects in trapping nanoparticles with femtosecond pulses. *Nat. Phys.*, 6(12):1005, 2010.

[4] Leiming Zhou, Yaqiang Qin, Yuanjie Yang, and Yuqiang Jiang. Fine features of optical potential well induced by nonlinearity. *Opt. Lett.*, 45(22):6266, 2020.

[5] J. R. Arias-González and M. Nieto-Vesperinas. Optical forces on small particles: attractive and repulsive nature and plasmon-resonance conditions. *J. Opt. Soc. Am. A*, 20(7):1201, 2003.

[6] Joseph Kestin, Mordechai Sokolov, and William A. Wakeham. Viscosity of liquid water in the range −8 °C to 150 °C. *J. Phys. Chem. Ref. Data*, 7(3):941–948, 1978.

[7] Robert L. Olmon, Brian Slovick, Timothy W. Johnson, David Shelton, Sang-Hyun Oh, Glenn D. Boreman, and Markus B. Raschke. Optical dielectric function of gold. *Phys. Rev. B*, 86(23):235147, 2012.

[8] Yuqiang Jiang, Yoshitaka Matsumoto, Yoichiroh Hosokawa, Hiroshi Masuhara, and Isamu Oh. Trapping and manipulation of a single micro-object in solution with femtosecond laser-induced mechanical force. *Appl. Phys. Lett.*, 90(6):061107, 2007.

[9] Étienne Boulais, Rémi Lachaine, and Michel Meunier. Plasma Mediated off-Resonance Plasmonic Enhanced Ultrafast Laser-Induced Nanocavitation. *Nano Lett.*, 12(9):4763–4769, 2012.
