# Supplementary material for: Nonlinearity-induced nanoparticle circumgyration at sub-diffraction scale
Source: Nat Commun. 2021 Jun 17;12:3722. doi: 10.1038/s41467-021-24100-0 (PMC8211862; doi:10.1038/s41467-021-24100-0)
Supplement: Supplementary file 2 — Description of Additional Supplementary Files [file 41467_2021_24100_MOESM2_ESM.docx]

Description of Additional Supplementary Files

Title: Supplementary Movie 1

Description: The motion of a GNP trapped by a circularly polarized femtosecond laser with right handedness. The Movie was taken at 1500 frames/s and replayed at a rate of 20 frames/s.

Title: Supplementary Movie 2

Description: The motion of a GNP trapped by a circularly polarized femtosecond laser with left handedness. The Movie was taken at 1500 frames/s and replayed at a rate of 20 frames/s.

Title: Supplementary Movie 3

Description: Front-view and side-view of a GNP motion. The Movie was taken at 1500 Hz and replayed at a rate of 20 frames/s.

Title: Supplementary Movie 4

Description: The motion of multiple GNPs trapped by a circularly polarized femtosecond laser with left handedness. The Movie was taken at 1500 Hz and replayed at a rate of 20 frames/s.
